# Comparison of self and simulated patient assessments of first-year medical students’ Interpersonal and Communication Skills (ICS) during Objective Structured Clinical Examinations (OSCE)

**DOI:** 10.1186/s12909-021-02540-y

**Published:** 2021-02-17

**Authors:** Joshua A. Roshal, Dalya Chefitz, Carol A. Terregino, Anna Petrova

**Affiliations:** Rutgers Robert Wood Johnson Medical School, Medical Education Building, MEB 202, 125 Paterson Street, New Brunswick, NJ 08901 USA

**Keywords:** Evaluation, Self-assessment, medical students, Communication skills, Objective structured clinical examination

## Abstract

**Background:**

Interpersonal and communication skills (ICS) are important core competencies in medical education and certification. In this study, we identified self- and simulated patient (SP)-reported ratings of US first-year medical students’ ICS and the influence of age and gender on performance appraisal during the Objective-Structured Clinical Examination (OSCE).

**Methods:**

OSCE participants, including 172 first-year medical students and 15 SPs were asked to evaluate the students’ ICS using the American Board of Internal Medicine–Patient-Satisfaction Questionnaire (ABIM–PSQ), electronically and via paper, respectively. Self- and SP-reported ratings of students’ ICS were presented as the median on a 5-point Likert-scale and as three categories defined as “good,” “very good,” and “inadequate.”

**Results:**

SPs assessed all 172 students in the OSCE, while 43.6% of students assessed their own performance. The majority of students and SPs evaluated the students’ ICS as very good. 23.3% of SPs and 5.3% of students rated the medical students’ ability to encourage patient question-asking and answer questions as inadequate (*P* <  0.002). Neither age nor gender influenced the medical students’ self-assessment of ICS. Female SPs assigned lower scores to students in regard to respecting patients and encouraging patient question-asking and answering. Older SPs was more likely to assign lower scores on all survey questions.

**Conclusions:**

In the present study, self- and SP-reported ratings of first-year medical students’ ICS were mainly “very good” with no influence of students’ age or gender. Older age and female gender among the SPs were associated with a reduction in SP-reported ratings of students’ ICS.

**Supplementary Information:**

The online version contains supplementary material available at 10.1186/s12909-021-02540-y.

## Background

The demonstration of effective interpersonal and communication skills (ICS) is one of the core competencies in both pre-and post-graduate medical education, [1] as well as physician certification [2]. Since its introduction in 1975, the Objective-Structured Clinical Examination (OSCE) has been generally accepted for measurement of medical students’ ICS [3–5]. Multiple studies have acknowledged the inadequate accuracy of existing tools in assessing medical students’ ICS during OSCEs [6–9]. For instance, a recent systematic review comments on the insufficient psychometric properties of 8 rating scales used in the assessment of medical students’ ICS during OSCEs [6]. No consensus exists among institutions on the employment of faculty examiners, simulated patients (SP), or both to evaluate medical students’ ICS [7]. An analysis of various checklists used in OSCEs identified fair to moderate agreement between raters in the assessment of medical students’ ICS, making comparisons of medical students within and across institutions difficult [7]. The quality of the different scales used in the OSCE is questionable, possibly because an acceptable level of adequate physician-patient communication has not been defined [10]. SPs participate in OSCEs and are an integral part of the curriculum in medical education [11]. Since there is an acceptable level of agreement between faculty examiners and SPs in the assessment of medical students’ ICS, SPs satisfaction scores may be a reliable indicator of medical students’ ICS [8]. Furthermore, the self-assessment of ICS by medical students has been identified as an important component in medical education and the development of self-directed learning skills [ 9, 12]. One study that compared medical students’ self-reported and SP- and observer-assigned OSCE checklist rating during an OSCE demonstrated that students scored their communication skills lower compared to observers or SPs in 2 out of 12 categories [13]. A study from Norway that used video recordings demonstrated poor concordance between self-reported scores by graduating medical students and SP-assigned scores in regard to ICS [9]. From the perspective of first-year medical students, reviewing video recordings of their interactions with SPs to self-assess their ICS was feasible, practical and informative [12]. Obtaining a better understanding about the ICS of medical students with limited clinical exposure could be essential for adjusting curricula to ensure appropriate ICS development prior to starting clinical rotations. Another recent study identified a need for the early introduction of simulation-based training to develop ICS in preclinical medical curricula [14]. First-year medical students have significantly lower positive attitudes toward ICS training and perceived confidence about communicating with patients than fourth-year medical students [15]. To the best of our knowledge, no published studies have investigated the differences between and influencing factors associated with ICS ratings provided by first year medical students themselves and SPs. The purpose of this study was to compare the self-reported and SP-assigned ratings of first-year medical student’ ICS during an OSCE and determine the influence of age and gender on performance appraisal. We utilized five questions from the American Board of Internal Medicine–Patient Satisfaction Questionnaire (ABIM-PSQ) [16] that were previously used to assess medical students’ ICS by the actual clinic patients in Japan [17, 18]. We assumed that ABIM-PSQ that was designed to survey actual patients could provide valuable information regarding ICS of medical students to explore the direction for improvement prior to clinical exposure. This research is the first that uses the ABIM-PSQ to identify and analyze SP-given ratings of first-year medical students’ ICS and self-assessed by students who participated in OSCE.

## Methods

In this cross-sectional study, we compared the self-reported and SP-assigned ratings of first-year medical student’ ICS and evaluated the effect of demographic covariates (age and gender) on the medical students’ and SPs’ responses. The survey protocol was approved as exempt from full review by the Rutgers Health Sciences Institutional Review Board because the investigation is based on the anonymous responses from the first-year medical students and SPs who participated in the OSCE (Protocol #2018002140).

### OSCE setting

The simulated clinical encounter was conducted in an OSCE setting with a 15-min time allotment for the medical student–SP interview. At our institution, the first-year medical students’ ICS in OSCEs are measured only by faculty. OSCE in the first-year medical students do not include ICS scoring by SPs as well as students themselves.

### Survey instruments

The ABIM–PSQ is a reliable and validated tool for evaluating global communication skills in physicians [16]. We used five questions (Table 1) [17, 18] from the original 10-item ABIM–PSQ because first-year medical students are only responsible for eliciting a patient history and performing a basic physical examination on SPs during the OSCE. Previous studies have suggested that the reliability of the ABIM–PSQ is not compromised as long at least five items from the original 10-item questionnaire are scored [16, 18]. As shown in Table 1, three questions measure patient-centered humanistic behavior, including greeting and friendliness (Q1), respect for patients (Q2), and careful listening (Q4). Other questions measure the personal interest displayed towards the SP (Q3) and encouragement of patient question-asking and answering questions (Q5). We used a 5-point Likert scale, where poor = 1, fair = 2, good = 3, very good = 4, and excellent = 5, to evaluate the medical students’ ICS according to each question-based statement. Finally, we asked medical students and SPs to indicate their age and gender. The questionnaire was uniform for medical students as well as SPs.

**Table 1:** Items selected from the ABIM–PSQ [17, 18]

Question (Q)	Please assess your own performance during the OSCE according to the following criteria:
**Q1** (Greeting and Friendliness)	Greeting you warmly; calling you by the name you prefer; being friendly; never crabby or rude.
**Q2** (Respect for Patients)	Treating you like you’re on the same level; never “talking down” to you or treating you like a child.
**Q3** (Personal Interest)	Letting you tell your story; listening carefully; asking thoughtful questions; not interrupting you while you’re talking.
**Q4** (Careful Listening)	Showing interest in you as a person; not acting bored or ignoring what you have to say.
**Q5** (Encouraging and Answering Questions)	Encouraging you to ask questions; answering them clearly; never avoiding your questions or lecturing you.

### Survey implementation

The questionnaire included informed consent that explained the goal and voluntary nature of this anonymous survey study along with the analogous questionnaires to the medical students and SPs electronically and via paper, respectively. We surveyed 172 first-year medical students and 15 SPs who had participated in the second OSCE of the first-year medical school curriculum but did not directly assess students’ ICS because at our institution, first-year medical students’ ICS in OSCEs are measured by faculty physicians. The member of the research team who distributed and collected the survey responses from the SPs did not participate in any OSCE-related activities. Moreover, no prior knowledge about the study was provided to SPs and medical students. Medical students and SPs were oriented to the expectations of the OSCE but did not have prior knowledge of the survey questions.

We provided an anonymous link to the survey questionnaire (https://rutgers.qualtrics.com) to the medical students on the day of the OSCE. Reminder messages were sent to all of the first-year medical students at 1 and 2 weeks from the original request. The SPs were asked to complete the paper-and-pencil survey for each student after testing during the OSCE, anonymously. Therefore, the self-assigned and SP-given ratings of first-year medical students’ ICS during the OSCE were not linked. The responses from SPs were collected at the end of the OSCE.

### Statistical analysis

We used the Mann-Whitney U test to compare the median scores on a 5-point Likert-scale, for the responses to each survey question, between the first-year medical students and SPs. Gender-driven differences in responses by medical students and SPs were calculated. We also used the *χ*^2^ test to compare scores on a 3-point Likert-scale, where responses were categorized based on a previous study [19] as “very good” if the medical students or SPs responded “excellent” or “very good”, “inadequate” if the medical students or SPs responded “poor” or “fair,” and “good” if the medical students or SPs ranked their answer as “good.” We also conducted a regression analysis to control the responses for the ages and genders of study participants (medical students and SPs). Categorical data are presented as percentages (%) and continuous data as means and standard deviations (SD), while Likert scale data are summarized by the medians and interquartile ranges (IQR). Regression coefficients (β) and odds ratios (OR) including 95% Confidence Interval (95%CI) were also used to present the results of the study. We used Statistica 13.2 for Windows (StatSoft Inc., Tulsa, OK) to analyze the data. A two-tailed *P*-value of less than 0.05 was considered statistically significant.

## Results

**Fig. 1:**
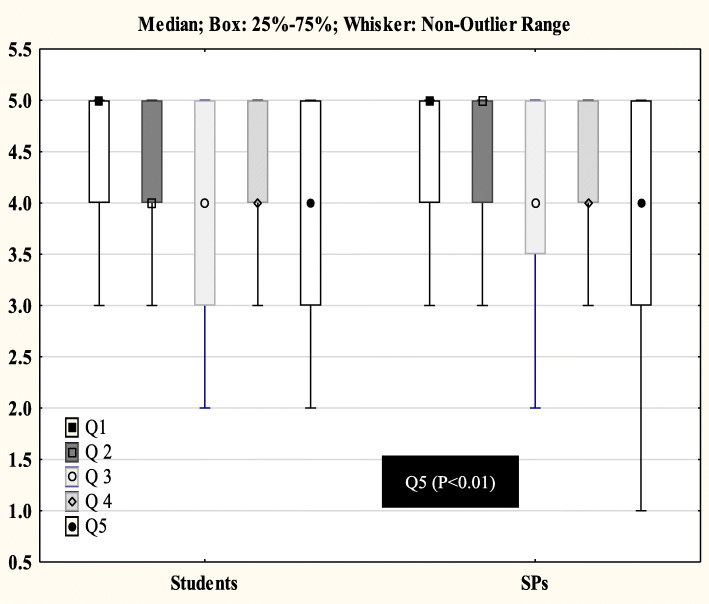
Comparison of median responses between medical students and simulated patients using a 5-point Likert scale

Among the 172 first-year medical students who were surveyed, 75 (43.6%) responded. SPs had evaluated between 9 and 15 first-year medical students (median = 12, IQR = 1) each. All respondents (first-year medical students and SPs) had completed the full questionnaire, except for one female medical student who did not identify her age. The ages of the first-year medical students and SPs ranged from 21 to 33 years (23.6 ± 2.1 years) and 25 to 60 years (46.0 ± 12.0 years), respectively (*P* <  0.0001). Male subjects constituted 40% of first-year medical students and 47.1% of SPs (*P* = 0.30). The median responses between the medical students and SPs using the 5-point Likert (Fig. 1) and 3-point Likert-scale (Table 2) were comparable except for question 5. As shown in Tables 2, 23.3% of the SPs assessed the medical students’ performance on question 5 as “inadequate” compared to 5.3% of medical students (*P* < 0.002).

**Table 2:** Comparison of medical students’ and simulated patients’ responses (% and 95 CI)

Question	Response	Group		P-value
		Students (***n*** = 75)	Patients (***n*** = 172)	
**Q1**	Inadequate	0	4.1 (1.7, 8.2)	0.14
	Good	9.3 (3.8, 18.3)	12.8 (8.2, 18.7)	
	Very Good	90.7 (81.7, 96.2)	83.1 (76.7, 99.4)	
**Q2**	Inadequate	0	1.2 (0.1, 4.1)	0.61
	Good	8.0 (3.0, 16.6)	9.3 (5.4, 14.7)	
	Very Good	92.0 (83.4, 97.0)	89.5 (84.0, 93.7)	
**Q3**	Inadequate	4.0 (0.8, 11.2)	4.1 (1.7, 8.2)	0.99
	Good	21.3 (12.7, 32.3)	20.9 (15.1, 27.8)	
	Very Good	74.7 (63.3, 84.0)	75.0 (67.8, 81.3)	
**Q4**	Inadequate	0	5.2 (2.4, 9.7)	0.12
	Good	13.3 (6.6, 23.2)	12.2 (7.7, 18.1)	
	Very Good	86.7 (76.8, 93.4)	82.6 (76.0, 87.9)	
**Q5**	Inadequate	5.3 (1.5, 13.1)	23.3 (17.2, 30.3)	< 0.002
	Good	20.0 (11.6, 39.8)	22.1 (16.1,29.0)	
	Very Good	74.7 (63.3, 84.0)	54.7 (46.9, 62.2)	

### Demographic characteristics and medical students’ and SPs’ responses

**Fig. 2:**
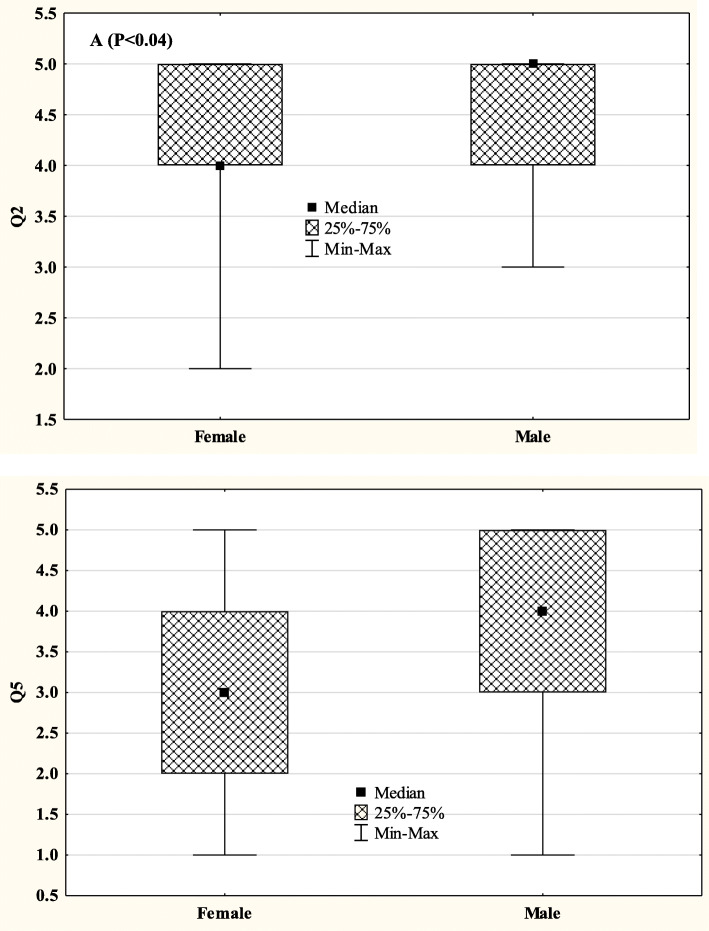
Gender differences in simulated patients’ responses to Q2 (**a**) and Q5 (**b**)

The medical students’ self-assessment scores were not associated with age. Gender did not influence the medical students’ responses (eTable 1 in Supplementary Material). However, female SPs were more likely to assign lower scores to the medical students on questions 2 and 5 as compared to their male counterparts (Fig. 2).These findings persisted even after controlling for the age of the SPs (Q2: OR 0.85, 95% CI 0.72–0.97 and Q5: OR 0.84, 95% CI 0.74–0.99). Irrespective of gender, older SPs were more likely to assign lower scores to the medical students on all survey questions as compared to their younger counterparts (Table 3).

**Table 3:** Association of simulated patients’ ages with responses (*P* < 0.0001)

Question	Coefficient (β)	95% CI (β)
**Q1** (Greeting and Friendliness)	−0.392	−0.531, −0.253
**Q2** (Respect for Patients)	−0.415	−0.553, −0.278
**Q3** (Personal Interest)	−0.497	− 0.628, − 0.366
**Q4** (Careful Listening)	− 0.498	−0.629, − 0.367
**Q5** (Encouraging and Answering Questions)	− 0.286	−0.432, − 0.141

## Discussion

This study found that the majority of first-year medical students and SPs evaluated the medical students’ greeting and friendliness, respect for patients, personal interest displayed towards the SP and careful listening during the OSCE as “very good.” However, students were more likely to overestimate their ability to encourage patient question-asking and answering questions. Neither age nor gender influenced the students’ self-assessment of their ICS. On the other hand, older SPs were more likely to assign lower scores to students. Furthermore, the female SPs assigned scores that were nearly 25% lower than the male SPs to medical students in regard to respecting patients and encouraging patient question-asking and answering questions. The discussion of findings in our study is limited. Previous studies have not investigated self-reported and SP-assigned ratings of first-year medical student’ ICS during an OSCE using the ABIM-PSQ. Only one study has reported self-assessed strengths and weaknesses of ICS by first-year medical students after viewing video recordings of their interactions with SPs [12]. More than 50% of students identified their ability to elicit information/cover important topics and personal connection/rapport as strengths but few students recognized weaknesses in their ICS, especially in non-verbal communication such as paralanguage, kinesics and facial expression. Several studies have discussed the ICS of medical students in their clinical years of medical school and echo the findings of our study. A study from Japan that used the ABIM-PSQ questionnaire demonstrated that actual patients were more likely to assign low scores to medical students during their clinical rotations in regard to encouraging patient question-asking and answering questions [18]. Meta-analyses that included 35 articles on medical student self-assessment accuracy defined with correlation, paired or independent means comparison revealed the tendency for students to overestimate their communication skill rather than knowledge-based assessments [20]. Unfortunately, age- and gender-based analyses of medical students’ self-reported ICS were rarely reported [20]. A longitudinal study from Germany demonstrated that female medical students in their 6th semester had higher self-reported empathy scores than their male counterparts. In the same study, female medical students were rated higher than their male counterparts by SPs on all dimensions tested during the OSCE, which included empathy, content structure, verbal expression, and non-verbal expression [21]. Another study from Japan found that observers assigned higher ratings of ICS to female fifth-year medical students compared to their male counterparts [22]. Furthermore, a study from the U.S. reported that SPs assigned significantly higher ratings of empathy displayed during an OSCE to female third- and fourth-year medical students compared to their male counterparts, irrespective of gender and ethnicity [23]. Berg et al. [24] found that female third-year medical students received higher scores on all three measures of empathy during an OSCE compared to their male counterparts. We found no studies that investigated the role of SP age of the assessment of medical students’ ICS.

This study has several limitations, including the external validity of findings from a sample of a single medical school. However, the distribution of genders in our study sample is comparable to that of all US first-year medical students [25]. There is also a risk of response bias since only 43.7% of first-year medical students responded to the survey, even though the distributions of the respondents’ ages and genders were comparable to the first-year medical student class at-large. Although complete anonymity increases response validity, it also could decrease the motivation to answer questions accurately [26, 27]. Since anonymity was preserved, we were not able to perform a paired analysis and instead relied on comparing the independent median scores, which is a statistically weaker measure of self-assessment accuracy [20]. Categorical data could improve the accuracy of the findings in our study [ 20]. However, determining the accuracy of our ICS measurements was not one of the goals of our study. We also recognize that collecting racial and ethnic data may be important for understanding the expectations of a culturally diverse patient population in regard to the ICS of future physicians. Moreover, we did not collect data as to whether the medical students successfully finished their SP encounters within the 15-min time limit. In a study of OSCE perspectives, third- and final-year medical students reported that the time allotted for OSCE stations involving medical interviews was insufficient. It is difficult to comment on the influence of time constraints on ICS because the OSCEs in these studies consisted of multiple stations that assessed clinical skills in addition to ICS [28, 29]. Nevertheless, the first-year medical students in our study may not have had the opportunity to encourage question-asking and answer questions due to time constraints. As a result, the disparity between self-assigned and SP-given ratings of performance in this domain may reflect an inability to finish the encounter on-time instead of a deficit in ICS.

## Conclusions

Despite the fact that most of the self-reported and SP-given ratings of first-year medical students’ ICS were “very good,” up to one-third of SPs defined the first-year medical students’ ability in encouraging patient question-asking and answering questions as “inadequate.” The influence of SP older age and female gender on the reduction of scores of medical students’ ICS, particularly in regard to encouraging patient question-asking and answering questions has been demonstrated. The findings of this study may have important implications for medical education, including curriculum development to ensure that first-year medical students are prepared to facilitate patient-centered communication and shared treatment-decision making during their clinical rotations [30]. Therefore, teaching pre-clinical medical students to encourage patient question-asking and answer questions may be important in advancing the ICS of future physicians.

## Supplementary Information

**Additional file 1 eTable 1.** Gender differences in medical students’ responses (Median and IQR).

## Data Availability

The datasets used and/or analyzed during the current study are available from the corresponding author on reasonable request.

## Abbreviations

OSCE: Objective Structured Clinical Examination(s)
SP: Standardized Patient(s)
ICS: Interpersonal and Communication Skills
ABIM-PSQ: American Board of Internal Medicine–Patient Satisfaction Questionnaire
RWJMS: Rutgers–Robert Wood Johnson Medical School
SD: Standard Deviation(s)
OR: Odds Ratio(s)
IQR: Interquartile Range(s)
CI: Confidence Interval(s)
