# Supplementary material for: Comparison of self and simulated patient assessments of first-year medical students’ Interpersonal and Communication Skills (ICS) during Objective Structured Clinical Examinations (OSCE)
Source: BMC Med Educ. 2021 Feb 17;21:107. doi: 10.1186/s12909-021-02540-y (PMC7887830; doi:10.1186/s12909-021-02540-y)
Supplement: Supplementary file 1 — Additional file 1 eTable 1. Gender differences in medical students’ responses (Median and IQR). [file 12909_2021_2540_MOESM1_ESM.docx]

**eTable 1.** Gender differences in medical students’ responses (Median and IQR)

| **Question** | **Medical Students** | | **P-value** |
| --- | --- | --- | --- |
|  | **Male (n=30)** | **Female (n=45)** |  |
| **Q1** |  |  | 0.14 |
|  | 4.0 (4-5) | 5.0 (4.0-5.0) |  |
|  |  |  |  |
| **Q2** |  |  | 0.21 |
|  | 4.0 (4.0-5.0) | 5.0 (4.0-5.0) |  |
|  |  |  |  |
| **Q3** |  |  | 0.63 |
|  | 4.0 (3.0-4.3) | 4.0 (4.0-5.0) |  |
|  |  |  |  |
| **Q4** |  |  | 0.57 |
|  | 4.0 (4.0-5.0) | 4.0 (4.0, 4.5) |  |
|  |  |  |  |
| **Q5** |  |  | 0.18 |
|  | 4.0 (3.0-5.0) | 4.0 (4.0- 5.0) |  |
|  |  |  |  |
